# Kernel Differential Subgraph Analysis to Reveal the Key Period Affecting Glioblastoma

**DOI:** 10.3390/biom10020318

**Published:** 2020-02-17

**Authors:** Jiang Xie, Jiamin Sun, Jiatai Feng, Fuzhang Yang, Jiao Wang, Tieqiao Wen, Qing Nie

**Affiliations:** 1 School of Computer Engineering and Science, Shanghai University, NanChen Road 333, Shanghai 200444, China; jiaminsun@i.shu.edu.cn (J.S.); fjtoo@shu.edu.cn (J.F.); crevenyoung@shu.edu.cn (F.Y.); 2 Laboratory of Molecular Neural Biology, School of Life Sciences, Shanghai University, Nanchen Road 333, Shanghai 200444, China; wtq@shu.edu.cn; 3 Department of Mathematics, the Center for Mathematical and Computational Biology, and the Center for Complex Biological Systems, University of California-Irvine, Irvine, CA 92697, USA; qnie@math.uci.edu

**Keywords:** glioblastoma, kernel differential subgraph, complex networks, single-cell, scRNA-seq

## Abstract

Glioblastoma (GBM) is a fast-growing type of malignant primary brain tumor. To explore the mechanisms in GBM, complex biological networks are used to reveal crucial changes among different biological states, which reflect on the development of living organisms. It is critical to discover the kernel differential subgraph (KDS) that leads to drastic changes. However, identifying the KDS is similar to the Steiner Tree problem that is an NP-hard problem. In this paper, we developed a criterion to explore the KDS (CKDS), which considered the connectivity and scale of KDS, the topological difference of nodes and function relevance between genes in the KDS. The CKDS algorithm was applied to simulated datasets and three single-cell RNA sequencing (scRNA-seq) datasets including GBM, fetal human cortical neurons (FHCN) and neural differentiation. Then we performed the network topology and functional enrichment analyses on the extracted KDSs. Compared with the state-of-art methods, the CKDS algorithm outperformed on simulated datasets to discover the KDSs. In the GBM and FHCN, seventeen genes (one biomarker, nine regulatory genes, one driver genes, six therapeutic targets) and KEGG pathways in KDSs were strongly supported by literature mining that they were highly interrelated with GBM. Moreover, focused on GBM, there were fifteen genes (including ten regulatory genes, three driver genes, one biomarkers, one therapeutic target) and KEGG pathways found in the KDS of neural differentiation process from activated neural stem cells (aNSC) to neural progenitor cells (NPC), while few genes and no pathway were found in the period from NPC to astrocytes (Ast). These experiments indicated that the process from aNSC to NPC is a key differentiation period affecting the development of GBM. Therefore, the CKDS algorithm provides a unique perspective in identifying cell-type-specific genes and KDSs.

## 1. Introduction

Glioblastoma (GBM) is one of the most common and lethal primary tumors, which has a poor prognosis and patients usually survive less than 15 months following diagnosis [1,2]. It is notoriously difficult to treat due to its diffuse nature and our limited knowledge of its molecular pathogenesis [3]. The important steps for determining the optimal therapeutic strategies are understanding the mechanisms of the dynamic processes and identification of new potential biological modules.

Compared with the bulk RNA sequencing, single-cell RNA sequencing (scRNA-seq) can provides important information for inter-cellular transcriptomic heterogeneity and dissecting the interplay between the cancer cells and the associated microenvironment. scRNA-seq is increasingly used to study gene expression at the level of individual cells and graduated processes such as development and differentiation, adding another dimension to understand gene expression regulation and dynamics [4]. Occurrence and development of cancers are governed by complex networks of interacting intercellular and intracellular signals [5,6]. 

Complex biological networks are able to reveal biological mechanisms [7]. Moreover, differential network is often used to identify the kernel modules causing diversity by integrating dynamic gene expression changes. Bai Zhang proposed the differential dependency network (DNN) method [8], which is based on local dependency, to detect topological changes across different biological conditions. A differential network-based methodology [9] can identify candidate target genes and chemical compounds for reverting disease phenotypes. BioNetStat is a tool for biological networks differential analysis by the methods grounded on network theory [10]. Furthermore, crucial changes among networks of different states are capable of reflecting on the development of living organisms [11]. Therefore, it is critical to discover the kernel differential subgraph (KDS) which leads to drastic changes. Discovering the KDS is similar to the Steiner Tree problem which is a NP-hard problem [12]. Topology-based KDS (TKDS) [13] is a method to discover the KDS from gene regulatory networks of omics datasets. SMT-Neurophysiology [12] is a tool in the form of an approximation to the Steiner Minimal Tree (SMT) algorithm, which is to find biomedically-meaningful KDS in neurophysiology. These methods could discover the KDS in different states. However, the accuracy of these methods was not high enough. And moreover, these methods did not fully consider the changes of topology.

The kernel differential subgraph (KDS) is a small-scale connected network with the differential nodes and edges. Considering the multiple factors affecting the subgraph, we developed a criterion to discover the kernel differential subgraph (CKDS). Specially, the criterion considers the connectivity and scale of KDS, the topological difference of nodes and function relevance between genes in the KDS. 

To demonstrate the effectiveness of our method, we applied CKDS to simulated datasets and three scRNA-seq datasets including GBM, fetal human cortical neurons (FHCN) and neural differentiation. Additional network topology and functional enrichment analyses were performed on the extracted KDSs influencing GBM closely.

## 2. Materials and Methods 

### 2.1. A Framework of a Criterion to Discover the Kernel Differential Subgraph (CKDS)

#### 2.1.1. Raw Data Pre-Treatment and Differential Expressed Genes Identification

**Figure 1:**
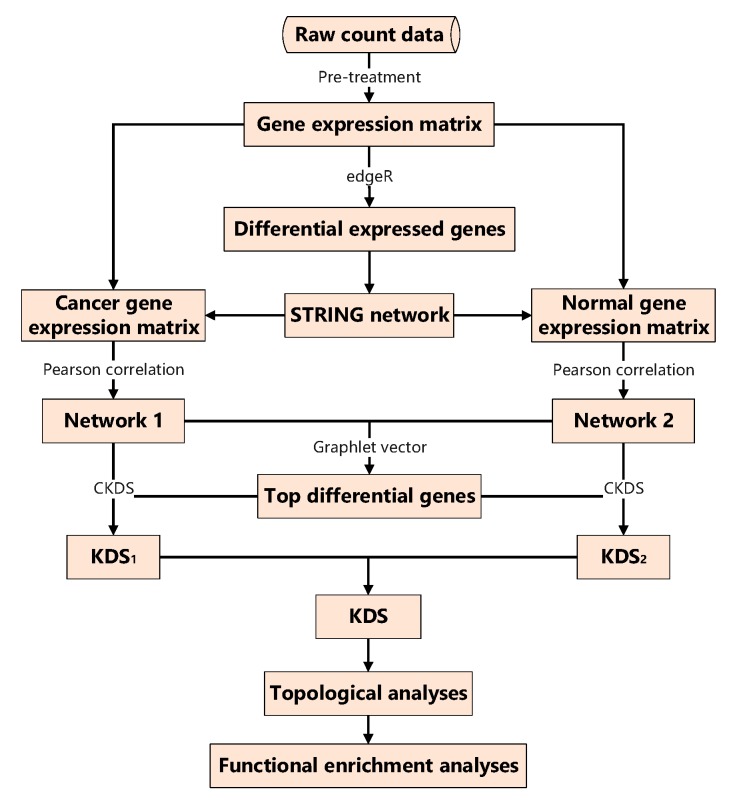
The overall framework for criterion to explore the kernel differential subgraph (CKDS). KDS: kernel differential subgraph.

Raw scRNA-seq counts data is usually downloaded from the Gene Expression Omnibus (GEO, http://www.ncbi.nlm.nih.gov/geo/). The raw counts data is converted to read-counts-per-million (CPM) gene expression matrix using the ‘cpm’ function by R package ‘edgeR’ [14]. The processed matrix is divided into cancer and normal gene expression matrix shown in Figure 1.

Differential expressed genes (DEGs) are detected from the processed scRNA-seq data by R package ‘edgeR’. Similar to the analysis of differential gene expression, we use ‘*p*-value, *p*.adjust and log_2_FC’ to obtain the DEGs by the gene expression. The R function ‘*p*.adjust’ is to adjust the p-values by ‘false discovery rate (fdr)’ method [15]. Fold change (FC) [16] is calculated simply as the ratio of the difference between final value and the initial value over the original value. In the field of bioinformatics, we commonly use log_2_ for expressing the FC (log_2_FC). The genes with the *p*-value < 0.01, *p*.adjust < 0.05 and $\left|log_{2}\mathrm{FC}\right|>2$ are considered as DEGs. 

#### 2.1.2. Single-Cell Transcriptome Network Construction by Differential Expressed Genes

Single-cell transcriptome data may lead to high false positives [17]. Therefore, integrated multi-omics data analysis has become a trend to solve it in biological network analysis [18]. Proteomics and transcriptomics data are integrated to construct a network [19], in which protein–protein networks (PPN) are used as a backbone network, and Pearson correlation coefficient (PCC) between expression of each pair of genes is used as the weight of edge. In this work, DEGs are connected with known protein–protein interactions (PPIs) documented in STRING database (v10.5, https://string-db.org/cgi/input.pl). Previous research has shown that when applied to real data, only edges with top 10% PCC were reserved [20]. In the generated STRING network, compared with the original one, over 90% edges disappear, and due to the generic property that the network structure would remain stable during the stable biological stage. At the same time, in order to ensure the effectiveness of PCC, we set |PCC|≥0.6 [21]. Thus, the association of two differential genes is defined as the weight of the edge, and only genes with the value of top 10% PCC and |PCC|≥0.6 are reserved.

#### 2.1.3. Calculating Differential Value of Genes by Graphlet Vector

**Figure 2:**
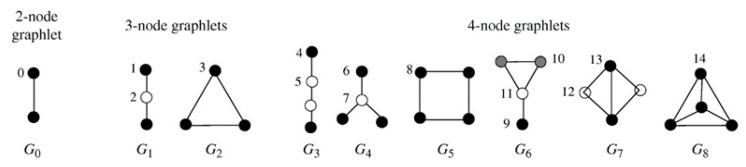
2–4-node graphlets G0–G8 and their automorphism orbits0–orbits14 [25].

Graphlets are small connected non-isomorphic induced subgraphs containing 2, 3, 4, or more nodes [22,23,24]. The graphlets of 2–4 nodes are shown in Figure 2. For 2, 3, and 4-node graphlets, the nodes in same color mean the nodes with the same topological structure (degree). There are 15 different kinds of nodes labelled orbits0-orbits14. Each node in the network obtains specific graphlet vector by calculating the frequency in 15 dimensions. 

For the node $u\in V,u^{\prime }\in V\prime ,$ $u_{i}$ denotes the *i^th^* coordinate of its signature vector, i.e. $u_{i}$ is the number of times node u is touched by an orbit *i* in V. The distance $D_{i}\left(u,u^{\prime }\right)$ between the *i^th^* orbits of nodes u and $u^{\prime }$ is defined as [25]:

(1) $$D_{i}\left(u,u^{\prime }\right)=w_{i}\times \frac{\left|\log\left(u_{i}+1\right)-\log\left({u^{\prime }}_{i}+1\right)\right|}{\log\left(\max\left\{u_{i},{u^{\prime }}_{i}\right\}+2\right)}$$

where $w_{i}$ is the weight of orbit *i* that accounts for dependencies between orbits [25].

As shown in Equation (2), the *d*-value between nodes u and $u^{\prime }$ means the total distance.

(2) $$d-\mathrm{value}\left(u,u^{\prime }\right)=\frac{\sum_{i=0}^{14}D_{i}\left(u,u^{\prime }\right)}{\sum_{i=0}^{14}w_{i}}$$

The distance $d-\mathrm{value}\left(u,u^{\prime }\right)$ is in (0, 1), where distance 0 means that signatures of nodes u and $u^{\prime }$ are identical [25]. The more topological structure varies, the larger *d*-value is. Nodes with *d*-value larger than 0.4 [23] are selected into the differential nodes set D for the further analysis.

#### 2.1.4. The Criterion to Extract Kernel Differential Subgraph

Kernel differential subgraph extraction is similar to Steiner Tree problem, which is an NP-hard problem. In this work, the criterion to extract KDS is present by four principles. Firstly, the subgraph should be connected. A connected subgraph can discover the dense relationship between molecules. Secondly, the scale of subgraph should be as small as possible. A KDS is the most core subgraph with small scale of the entire network. Thirdly, the *d*-value of nodes with large topological difference calculated by graphlet should be as large as possible. Nodes with large differences in topology are often key nodes in the network. These nodes will be selected to extract the KDS. Fourthly, the functional relevance between genes should be as strong as possible. It means the higher weight of edges will be chosen to extract the KDS.

There is a cancer network G(V,E) and a normal network G′(V,E′) representing two different states. V represents the set of *v* common nodes; E and E′ represent the set of edges respectively. Algorithm 1 describes the criterion to discover the KDS (CKDS), where $W_{e}$ represents the weight set of edges. The set $D=\left\{D_{1},D_{2},\ldots ,D_{v}\right\}$ represents the set of differential nodes with *d*-value $d=\left\{d_{1},d_{2},\ldots ,d_{v}\right\}$. According to the sorted *d*-value d in descending order, we selected the differential nodes $D_{v}$ ($d_{v}\geq 0.4$) [23] to add in KDS. When considering a new path added to KDS, $\sum d_{v}$ and $\sum W_{e}$ mean the sum of *d*-value of all nodes and weight of all edges on the path. The parameter a and b were coefficient designed to measure the importance of $\sum D_{v}$ and $\sum W_{e}$. The estimation of the vector (a,b) is discussed in Section 3. $KDS_{x}\;$($KDS_{1}$ and $KDS_{2})$ indicates the KDS of different state. The pseudo code of this algorithm is shown below.
Algorithm 1: the criterion to discover the KDS (CKDS) Input: network G, network G′, common differential node set $D=\left\{D_{1},D_{2},\ldots ,D_{v}\right\}$ with their *d*-value $d=\left\{d_{1},d_{2},\ldots ,d_{v}\right\}$. Output: KDS of G and G′. Sort D by their *d*-value d in descending order For x from 1 to 2   Add $D_{1}$ to $KDS_{x}$
  For $D_{v}\left(v\geq 2\;and\;d_{v}\geq 0.4\right)$ in sorted D do     if $D_{v}$ is existed in $KDS_{x}$
  continue     else if       if $D_{v}$ directly connect with any node existed in $KDS_{x}$
        add $D_{v}$ and its edge to $KDS_{x}$
      else if        calculate the score of the shortest paths from $D_{v}$ to each node in $KDS_{x}$ by       Equation (3),
(3)$$Score_{path}=a*\sum d_{v}+b*\sum W_{e}$$
      Add the path that has the highest $Score_{path}$ to $KDS_{x}$
  End   Return $KDS_{x}$
End Intersect $KDS_{1}$ and $KDS_{2}$ to get KDS of G and G′


After getting the KDS of G and G′, the KDS was constructed by Cytoscape (http://www.cytoscape.org/) [26]. 

### 2.2. Topological Analyses on Kernel Differential Subgraph

The centrality indexes including degree centrality (DC) [27], betweenness centrality (BC) [28], closeness centrality (CC) [29], and eigenvector centrality (EC) [30] were used to analyze the KDS. For a KDS G=(V,E), V and E represent the set of nodes and edges respectively. Four centrality indexes are defined as follows,

**DC:** DC means how many nodes connected to node v, and it can measure node v’s centrality apparently. $\left|N_{v}\right|$ is the number of node v’s neighbors. The degree of node v is formalized by Equation (4), where

(4) $$C_{D}\left(v\right)=\left|N_{v}\right|$$

**BC**: BC is the average length of the shortest paths through node  v. Equation (5) is as follow:

(5) $$C_{B}\left(v\right)=\underset{s\neq v\neq t\in V}{\sum }\frac{\sigma_{st}\left(v\right)}{\sigma_{st}}$$

In which, $\sigma_{st}$ is the total number of shortest paths from node s to node t. $\sigma_{st}\left(v\right)$ means the number of those paths that go through node v.

**CC**: In the network *V* with n nodes, closeness centrality means the degree that node v communicates with other nodes set $t=\left\{t_{0},t_{1},\ldots ,t_{m}\right\},0\leq \;m\leq n-1$. It is calculated by Equation (6):

(6) $$C_{c}\left(v\right)=\frac{n-1}{\sum_{m=0}^{n-1}dist\left(v,t_{m}\right)}$$

dist(v,t) is the distance of the shortest path from node v to node $t_{m}$.

**EC**: EC is a measure of the influence of node v on a network. It assigns relative scores to all nodes in the network based on the concept that connections to high-scoring nodes contribute more to the score of the node in question than equal connections to low-scoring nodes [30]. The EC score of node v is shown as Equation (7):

(7) $$C_{E}\left(v\right)=\alpha_{\max}\left(v\right)$$

$\alpha_{\max}$ is the eigenvector corresponding to the largest eigenvalue from *A* which is the adjacency matrix of KDS.

Different topology analysis methods rely on different network topology structures, which may not comprehensively balance the importance of genes in different biological states. Therefore, we employed four centrality indexes (one local measurement method ’DC’ and three global measurement methods ‘BC, CC, and EC’). According to four centrality indexes, four scores of each node in the subgraph was calculated and normalized to the number in the range 0 to 1. Each node would have a score to evaluate the topological differences in Equation (8). Multiple centralities can be considered comprehensively to evaluate the node topology.

(8) $$Score_{T}\left(v\right)={C^{\prime }}_{D}\left(v\right)+C_{B}^{\prime }\left(v\right)+{C^{\prime }}_{c}\left(v\right)+{C^{\prime }}_{E}\left(v\right)$$

${C^{\prime }}_{D}\left(v\right),\;C_{B}^{\prime }\left(v\right),\;{C^{\prime }}_{c}\left(v\right),\;{C^{\prime }}_{E}\left(v\right)$ means four normalized centrality indexes of node v. In the following study, we focused on the nodes with top 10% score, which were with large topological differences in the KDS.

### 2.3. Functional Enrichment Analyses

The Gene Ontology (GO) and the Kyoto Encyclopedia of Genes and Genomes (KEGG) analyses were performed to understand the underlying biological mechanisms. GO analyses explored the biological significance of genes by R package ‘clusterProfiler’ [31]. The enriched GO terms with Gene-Count > 5 and *p*-value < 0.05 were selected for further assessment [32]. In this paper, we also focused on the top 10% frequently occurring genes in the GO terms. The KEGG analyses were performed on pathways with *p*-value < 0.05. 

### 2.4. Evaluation Indicators

The number of essential genes in the KDS could evaluate the performance of the algorithm. The more essential genes were found, the better the performance of the algorithm was.

(9) $$P_{KDS}=\frac{N_{p}}{N_{e}}=\frac{N_{p}}{N_{p}+N_{p\prime }}$$

As shown in Equation (9), $P_{KDS}$ is calculated to evaluate the performance. $N_{e}$ means the number of essential genes.$N_{p}$ is the number of essential genes in KDS, and $N_{p\prime }$ is the number of essential genes which are not predicted in KDS. $P_{KDS}$ is similar to the evaluation indicator ‘Precision’ in binary classification problem.

To better evaluate the performance, true negative (TN), false positive (FP), false negative (FN), and true positive (TP) [33] are used to calculate evaluation indicators, including Accuracy, Precision ($P_{KDS}$), Recall, and F1-Score as following.

(10) $$Accuracy=\frac{TP+TN}{TP+TN+FP+FN}$$

(11) $$PrecisionP_{KDS}=\frac{TP}{TP+FP}$$

(12) $$Sensitivity=Recall=\frac{TP}{TP+FN}$$

(13) $$F_{1}=\frac{2*Precision*Recall}{Precision+Recall}$$

Moreover, F1-Score is a handy indicator for measuring the accuracy of a binary classification model. F1-Score takes Precision and Recall into account, which ranges from 0 to 1. The algorithm is more excellent if the F1-Score is closer to 1.

## 3. Results and Discussion

### 3.1. Simulated Data Generation

According to the principles of biomolecular network [34], we used a simulated data generating algorithm [35] to generate simulated data. 

The algorithm could generate two networks with a list of essential genes and two sets of gene expression based on some parameters. The parameters of $n_{1}$ and $n_{2}\;$ mean the number of nodes, and m means the number of essential genes in the two networks. The parameter 𝜌 means the proportion of differential edges driven by perturbed genes [35]. The smaller 𝜌 is, the more difficult it is to find essential genes. In this paper, $n_{1}=n_{2}=100$, m=10, ρ=0.1.

Two hundred groups of simulated datasets were generated, in which 100 groups (Dataset I) were to get the vector (a,b) in Equation (3) and 100 groups (Dataset II) were to compare the performance of CKDS with other methods. 

As the Equation (3) shows, the score of path is influenced by $\sum d_{v}$ and $\sum W_{e}$, and parameter a andb were designed to measure the importance of $\sum d_{v}$ and $\sum W_{e}$. In order to distinguish which variable is more influential, the sum of a andb was designed to be 1. For each of the 100 groups (Dataset I), the parameter a andb were taken from 0 to 1 respectively. Thus, the optimal ratio of a andb can be generated by conducting experiments on resulted KDS’s prediction precision by Equation (11).

**Figure 3:**
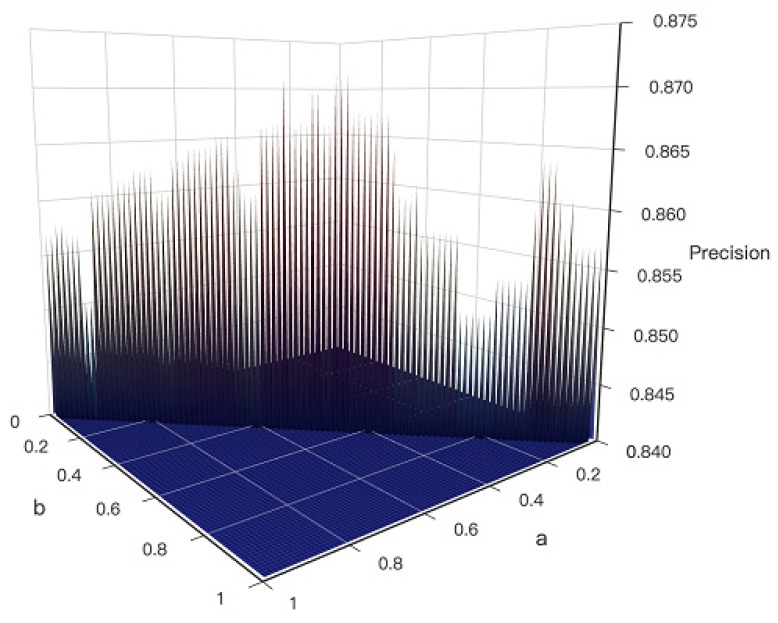
Three-dimensional **(**3D) Surface Graph of the result of Dataset I. The *x*-axis and *y*-axis represent the value of a and b respectively, and the *z*-axis represents the value of evaluation indicator precision ($P_{KDS}$).

As shown in Figure 3, the parameter a andb around 0.5 (a:b=1:1) gets the KDS with the highest Precision ($P_{KDS})$. It reflects that $\sum d_{v}$ is as important as $\sum W_{e}.$ According to the four principles, the CKDS algorithm considers the connectivity and scale of KDS, the topological difference of nodes and function relevance between genes in the KDS. The reason why the ratio of *a* to *b* is 1:1 is that when a new shortest path is added to the KDS, the ratio of the number of the points and edges is 1:1. The newly shortest added path meets four principles very well. The value of $d_{v}$ and $W_{e}$ are between 0 and 1 respectively. The ratio of $\sum d_{v}$ to $\sum W_{e}$ is close to 1. 

The experiment results showed that when the ratio of a andb is about to 1, the generated KDS can perform well and acquire the reliable result. According to the four principles of CKDS, the final equation to calculate the path in this paper is shown as Equation (14).

(14) $$Score_{path}=\sum d_{v}+\sum W_{e}$$

### 3.2. Comparison with other Methods on Simulated Datasets

In our work, we compared CKDS with other three differential kernel subgraph extraction algorithms: SMT-Neurophysiology (KDS-SMT) [12], TDKS [13] and KDS based on Floyd (KDS-Floyd) [36]. Each algorithm would get a KDS with essential genes. One hundred groups of simulated datasets(Dataset II) with 10 essential genes were generated to assess the performance of the four algorithms. 

After calculating the evaluation indicators by Equations (10)–(13), the results show CKDS is superior to other three algorithms on those measures (Table 1). It proves that CKDS has a good performance to find KDS with essential genes. This is because that CKDS combines multiple principles, which is capable of taking various kinds of differences into consideration.

### 3.3. The Kernel Differential Subgraph Analyses for Single-Cell RNA-Seq Datasets of Glioblastoma

#### 3.3.1. Single-Cell RNA-Seq Datasets of Glioblastoma and Fetal Human Cortical Neuron

The raw scRNA-seq data was downloaded from the GEO database. To compare GBM and normal cells, 134 fetal human cortical neurons (FHCN) [37] (GSE67835, 25 June, 2019) and 3589 human glioblastoma cells from Darmanis et al [38] (GSE84465, 25 June, 2019) were downloaded to discover the KDS between two states.

**Figure 4:**
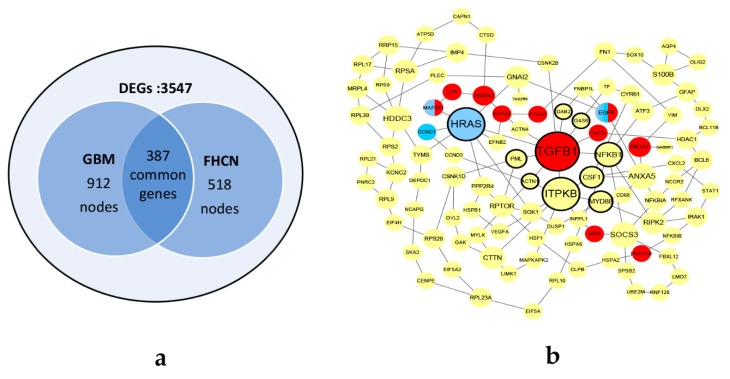
The datasets and KDS of glioblastoma (GBM) and fetal human cortical neurons (FHCN). (**a**) The pre-treatment datasets of GBM and FHCN. (**b**) The KDS of GBM and FHCN. The bolded border indicates the genes with high topological differences. The genes marked in red are frequently occurring in Gene Ontology (GO) terms. The genes marked in blue are enriched in glioma pathway by KEGG enrichment analysis. The half blue half red nodes indicate that the genes occur frequently in GO terms and are enriched in glioma pathway by Kyoto Encyclopedia of Genes and Genomes (KEGG) enrichment analyses.

Using two scRNA-seq datasets, differential expressed analyses were performed by ‘egdeR’ [14]. As shown in Figure 4a, 3547 genes were defined as DEGs. Two networks were constructed by the method illustrated in Section 2.1.2. The GBM network consists of 912 nodes with 1986 edges and the FHCN network consisted of 518 nodes with 594 edges. There were 387 common genes in two networks. The common genes were sorted by calculating the graphlet vector in descending order. Finally, using the CKDS algorithm, the KDS of GBM and FHCN was discovered, consisting of 106 genes with a total of 141 interactions in Figure 4b.

#### 3.3.2. The Analyses of Kernel Differential Subgraph

In order to explore the biological mechanisms of GBM, we used network topology and functional enrichment analysis methods on the extracted KDS. However, there is no golden standard in evaluate KDSs in real bio-network. In this paper, the effectiveness of the method can be accessed by literature mining.

According to four centrality indexes, each node in KDS was calculated by Equation (6). We focused on the top 10% nodes with the highest score in KDS. Eleven genes with large topological differences (*TGFB1*, *ITPKB*, *HRAS*, *NFKB1*, *PML*, *MYD88*, *ACTN1*, *CSF1*, *GAS6*, *DAB2* and *CSNK2B*) were chosen from the KDS of GBM and FHCN in Figure 4(b). Eight of the eleven genes were supported by the literature arguing that they had great influence on GBM. Among them, *TGFB1*, *PML* and *GAS6* are therapeutic targets for GBM. *NFKB1*, *CSF1* and *LYN* are regulatory genes which facilitate progression of GBM. *MYD88* is a biomarker to divide GBM patient. *ACTN1* is regulated during the development of astrocytoma cells. *HRAS* is a driver gene that expression of oncogenic *HRAS* results in a malignant phenotype in glioma cell lines (Table 2). 

The eleven genes that top 10% frequently occurred in the enriched GO terms were selected from GBM and FHCN and marked in red in Figure 4(b). Supported by the literature, ten of the eleven genes had great influence on GBM. Among them, *EGFR*, *DAXX*, *ANXA1*, *ANXA2* and *LYN* are regulatory genes which promote glioma growth. *HSPA1B*, *EPHA3*, *INSR* and *TGFB1* are functional therapeutic targets in glioblastoma (Table 3). *MAP2K1* is enriched in the KEGG pathway(hsa05214) for GBM.

By KEGG enrichment analysis, there was an enriched KEGG pathway (hsa05214: *HRAS*, *MAP2K1*, *EGFR* and *CCND1*) for GBM. 

In summary, by the topology and functional enrichment analyses on the KDS, seventeen genes (nine regulatory genes, six therapeutic targets, one driver gene, one biomarker) and one pathway were found, which were closely interrelated with GBM. The experiments indicated that the KDS extracted by CKDS reflected the large differences between GBM and FHCN, which highly influenced on the development of GBM.

### 3.4. The Kernel Differntial Subgraph Analyses for Single-Cell RNA-Seq Datasets of Neural Differentiation

#### 3.4.1. Single-Cell RNA-Seq Datasets of Neural Differentiation

To further explore the effects of neurodevelopmental stages and the development of GBM, the raw scRNA-seq data of neural differentiation about neural stem cell lineages from adult mice, including 152 activated neural stem cells (aNSCs), 64 produce neural progenitor cells (NPCs) and 31 astrocytes (Asts) were downloaded from the reference [55]. Three different stages of neural stem cell lineage are divided to Group A (aNSCs and NPCs) and Group B (NPCs and Asts).

**Figure 5:**
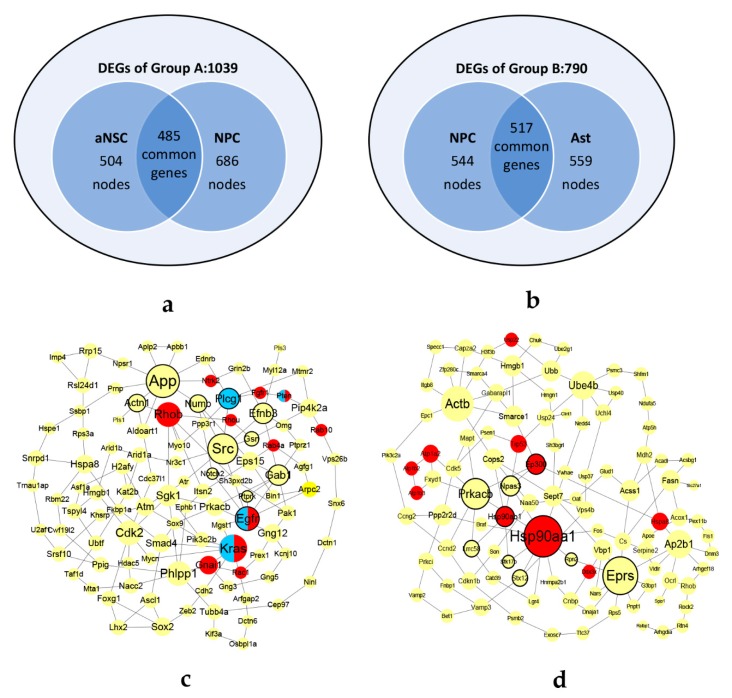
The datasets and KDSs of neural differentiation. The pre-treatment datasets of Group A (**a**) and Group B (**b**). The KDSs of Group A (**c**) and Group B (**d**). In (**c**) and (**d**), the bolded border indicates the genes with high topological differences. The genes marked in red are frequently occurring in GO terms. The genes marked in blue are enriched in Glioma pathway by KEGG enrichment analyses. The half blue half red nodes indicate that the genes occur frequently in GO terms and are enriched in Glioma pathway by KEGG enrichment analyses.

Differential expressed analysis was performed by *‘*egdeR’ packages. 1039 DEGs and 790 DEGs were extracted from two groups respectively (Figure 5a,b). The networks were constructed by Section 2.1.2. In Group A, the aNSCs network consisted of 504 nodes with 1492 edges and the NPCs network consisted 686 nodes with 2682 edges. In Group B, the NPCs network consisted of 544 nodes with 2686 edges and the Asts network consisted of 559 nodes with 2724 edges. There were 485 and 517 common genes in two groups respectively. The common genes in each group were sorted by calculating the graphlet vector in descending order. 

Using the CKDS algorithm, two KDSs of the two groups were discovered, consisting of 107 genes with 151 interactions in KDS-A and 109 genes with 144 edges in KDS-B, as shown in Figure 5c,d.

#### 3.4.2. Kernel Differential Subgraph Analyses

In Group A, according to four centrality indexes, top 10% genes with large topological differences in KDS-A was calculated by Equation (6). Eleven genes (*Src*, *Egfr*, *Gab1*, *App*, *Numb*, *Plcg1*, *Efnb3*, *Ptprk*, *Actn1*, *Notch2* and *Gsn*) were chosen from the KDS-A. The border lines of these genes are bolded in Figure 5c. Supported by the literature (Table 4), eight of the eleven genes have influence on GBM. Among them, *Src* is a driver gene which inhibit the growth of GBM and reduce its survival. *Egfr*, *Gab1*, *App* and *Efnb3* are regulatory genes which promote glioma cell proliferation. *Numb* has effective anti-cancer therapy in glioblastoma. *Plcg1* induces GBM radioresistance. *Notch2* and *miR-181a* have potential prognostic value as tumor biomarkers in GBM patients. 

Compare with Group A, only few genes (*Hsp90aa1*, *Eprs* and *Hsp90ab1*) supported by the literature references in KDS-B which have influence on GBM (Table 5).

From aNSCs to NPCs stage, top 10% frequently occurring genes in the enriched GO terms (*Rab4a*, *Pten*, *Egfr*, *Rab10*, *Rac1*, *Fgfr1*, *Gnai1*, *Ntrk2*, *Rhob*, *Kras* and *Rhou*) were selected to look for the biomarkers. These 11 gene nodes are marked in red in Figure 5c. Supported by the literature, eight of the eleven genes have influence on GBM. Among them, *Pten* and *Rac1* are driver genes which inhibit the migration and invasion of GBM. *Egfr*, *Kras*, *Gnai1*, *Ntrk2* and *Rhob* are regulatory genes which drive the initiation and progression of glioma. *Fgfr1* induces GBM Radioresistance. In KDS-B, seven genes (*Hsp90aa1*, *Hsp90ab1*, *Atp1b2*, *Trp53*, *Hspa8*, *Usp22* and *Atp1a2*) are supported by the literature references which have influence on GBM (Table 6 and Table 7). 

By KEGG enrichment analyses, the KDS enriched lots of KEGG pathways related to cancer, particularly, the KEGG pathway mmu05214 (*Egfr*, *Plcg1*, *Kras* and *Pten*) is exactly the pathway of GBM. 

In summary, the KDS-A involved ten regulatory genes, three driver genes, one biomarkers, one therapeutic target of GBM. These fifteen genes and the KEGG pathway in KDS-A highly influenced on the development of GBM. However, there was few genes and no pathway of GBM in KDS-B. 

The topological and functional enrichment analyses indicated the genes and pathways associated with glioma and cancers are significantly reduced during the period from NPC to Ast. It suggests that the critical period of GBM development is from aNSC to NPC other than NPC to Ast. 

Gliomas are malignant primary tumors of the central nervous system. Their cell-of-origin is thought to be a neural progenitor or stem cell that acquires mutations leading to oncogenic transformation [76]. By the CKDS algorithm, we proved that the stage of aNSCs to NPCs is a critical period affecting the development of GBM.

## 4. Conclusions

Complex biological networks are used to explore the mechanisms in complex diseases. Crucial changes in different networks reflect on the development of living organisms. Therefore, it is significant to discover the KDS leading to drastic changes.

In this work, we developed a criterion to discover KDS called CKDS. The criterion fully considered the factors affecting KDS, including the connectivity and scale of KDS, the topological difference of nodes and function relevance between genes in the KDS. As a result, the CKDS algorithm discovered the KDS in different states.

The CKDS algorithm was applied to simulated datasets and three scRNA-seq datasets including GBM, FHCN, and neural differentiation. Compared with the other state-of-art methods, the CKDS algorithm outperformed in simulated datasets to discover the KDSs. In the scRNA-seq datasets, we performed the network topology and functional enrichment analyses on the extracted KDSs. Many genes, including genetic biomarkers, driver genes, regulatory genes, and therapeutic targets, and pathways in the KDSs are closely interrelated to GBM, indicating that CKDS could express the kernel difference between different states. Moreover, the KEGG pathway of GBM is only in neural differentiation period from aNSC to NPC other than NPC to Ast, indicating that the period from aNSC to NPC is an important neural differentiation period affecting the development of GBM. In addition, the CKDS algorithm provides a unique perspective in identifying cell-type-specific genes and KDSs.

Based on the prediction of CKDS, the genes that were not supported by literature will be verified by conducting a series of biological experiments in the future. Moreover, the CKDS algorithm can be extended to scRNA-seq datasets of other complex diseases for detecting the molecular features of pathogenesis mechanisms and biomarkers.

## Figures and Tables

**Table 1:** The evaluation indicators of three classical methods compared with CKDS. TKDS: Topology-based KDS; KDS-SMT: kernel differential subgraph-Steiner Minimal Tree.

	Methods	KDS-SMT	KDS-Floyd	TKDS	CKDS
Indicators					
Accuracy		87.51%	81.30%	83.72%	88.86%
Precision ($P_{KDS}$)		0.684	0.793	0.797	0.871
Recall		42.30%	32.29%	35.87%	46.93%
F1-Score		0.523	0.459	0.495	0.610

**Table 2:** The biological functions, corresponding PubMed IDs and literatures for genes with large topological changes between GBM and FHCN.

Symbol: Gene Name	Function Roles in GBM	PMID Reference
*TGFB1*: transforming growth factor beta 1	the oncogenic *MSH6-CXCR4-TGFB1* feedback loop is a novel therapeutic target for GBM	30867843 [39]
*HRAS*: HRas proto-oncogene, GTPase	expression of oncogenic HRAS results in a malignant phenotype in glioma cell lines	27834733 [40]
*NFKB1*: nuclear factor kappa B subunit	increase glioma cancer risk	30450997 [41]
*PML*: promyelocytic leukemia	a *PML/SLIT1* axis regulates sensitivity to the *PML*-targeting drug arsenic trioxide in primary GBM cells	28700942 [42]
*MYD88*: MYD88 innate immune signal transduction adaptor	divide GBM patient	29168084 [43]
*ACTN1*: actinin alpha 1	influence the development of astrocytoma cells	20156433 [44]
*CSF1*: colony stimulating factor 1	*CSF1* signaling is oncogenic during gliomagenesis through a mechanism distinct from modulating GAM polarization status.	27013192 [45]
*GAS6*: growth arrest specific 6	represent a potential new approach for glioma treatment	18172262 [46]

**Table 3:** The biological functions, corresponding PubMed IDs and literature references for enriched genes by GO enrichment analyses between GBM and FHCN.

Symbol: Gene name	Function roles in GBM	PMID Reference
*EGFR*: Epidermal growth factor receptor	promote glioma growth and angiogenesis	22139077 [47]
*DAXX*: death domain associated protein	targeting telomerase and *ATRX/DAXX* inducing tumor senescence and apoptosis in the malignant glioma	30625996 [48]
*ANXA1*: Annexin A1	enhance cancer growth and migration	29263330 [49]
*ANXA2*: Annexin A2	affect the proliferation of human glioma cells through the *STAT3* cyclin D1 pathway via direct interaction with *STAT3* in U251 and U87 glioma cells	31115554 [50]
*LYN*: LYN proto-oncogene, Src family tyrosine kinase	facilitate glioblastoma cell survival under conditions of nutrient deprivation by promoting autophagy	23936469 [51]
*HSPA1B*: heat shock protein family A (Hsp70) member 1B	therapeutic targets for enhancing the efficacy of erlotinib against GBMs	19301967 [52]
*EPHA3*: EPH receptor A3	a functional tumour-specific therapeutic target in glioblastoma	30562956 [53]
*INSR*: insulin receptor	activation of the *InsR/IGF1R* pathway confers resistance to *EGFR* inhibitors in *EGFR*-dependent glioblastoma through AKT regulation	26561558 [54]
*TGFB1*: transforming growth factor beta 1	the oncogenic *MSH6-CXCR4-TGFB1* feedback loop is a novel therapeutic target for GBM	30867843 [39]

**Table 4:** The biological functions, corresponding PubMed IDs and literatures for genes with large topological changes between activated neural stem cell (aNSC) and neural progenitor cells (NPC).

Symbol: Gene Name	Function Roles in GBM	PMID References
*Src*: SRC proto-oncogene, non-receptor tyrosine kinase	Reduce human glioma stem cell migration, invasion, and survival	28712848 [56]
*Egfr*: Epidermal growth factor receptor	Promote glioma growth and angiogenesis	22139077 [47]
*Gab1*: GRB2 associated binding protein 1	Promote glioma cell proliferation	30016785 [57]
*App*: amyloid beta precursor protein	Promote the proliferation of glioma cells to inhibit the differentiation of glioma cells	28789439 [58]
*Numb*: NUMB endocytic adaptor protein	Effective anti-cancer therapy	31116627 [59]
*Plcg1*: phospholipase C gamma 1	Induce Glioblastoma Radioresistance	26896280 [60]
Efnb3: ephrin B3	Support glioblastoma growth	28423606 [61]
*Notch2*: notch receptor 2	Tumor biomarkers in GBM	28389242 [62]

**Table 5:** The biological functions, corresponding PubMed IDs and literatures for genes with large topological changes between NPC and astrocytes (Ast).

Symbol: Gene Name	Function Roles in GBM	PMID Reference
*Hsp90aa1*: heat shock protein 90 alpha family class A member 1	survival signatures in GBM	22952576 [63]
*Eprs*: glutamyl-prolyl-tRNA synthetase	the protein coding genes in GBM	30572911 [64]
*Hsp90ab1*: heat shock protein 90 alpha (cytosolic), class B member 1	predict prognosis in astrocytic tumors	27258564 [65]

**Table 6:** The biological functions, corresponding PubMed IDs and literature references for enriched genes by GO enrichment analyses between aNSC and NPC.

Symbol: Gene Name	Function Roles in GBM	PMID Reference
*Pten*: phosphatase and tensin homolog	Reduce human glioma stem cell migration, invasion, and survival	28712848 [56]
*Egfr*: Epidermal growth factor receptor	Promote glioma growth and angiogenesis	22139077 [47]
*Rac1*: Rac family small GTPase 1	Inhibit the migration and invasion of glioma	28714015 [66]
*Fgfr1*: fibroblast growth factor receptor 1	Induce Glioblastoma Radioresistance	26896280 [60]
*Gnai1*: G protein subunit alpha i1	The growth of subcutaneous and orthotopic glioma xenografts	29520106 [67]
*Ntrk2*: neurotrophic tyrosine kinase, receptor, type 2	Promote tumor growth	29625067 [68]
*Rhob*: ras homolog family member B	Differential implication of Rho GTPases in morphology, proliferation rate and motility of human glioblastoma cells	26741994 [69]
*Kras*: KRAS proto-oncogene, GTPase	Drive the initiation and progression of glioma	30946839 [70]

**Table 7:** The biological functions, corresponding PubMed IDs and literature references for enriched genes by GO enrichment analyses between NPC and Ast.

Symbol: Gene Name	Function Roles in GBM	PMID Reference
*Hsp90aa1*: heat shock protein 90 alpha family class A member 1	Survival signatures in GBM	30572911 [63]
*Hsp90ab1*: heat shock protein 90 alpha (cytosolic), class B member 1	predict prognosis in astrocytic tumors	27258564 [65]
*Atp1b2*: ATPase Na+/K+ transporting subunit beta 2	Na⁺/K⁺-ATPase β2-subunit (AMOG) expression abrogates invasion of glioblastoma-derived brain tumor-initiating cells.	23887941 [71]
*Trp53*: tumor protein p53	Induce G1/S phase cell cycle arrest in glioblastoma cells	31001122 [72]
*Hspa8*: heat shock protein family A (Hsp70) member 8	Inhibition of nestin suppresses stem cell phenotype of glioblastomas	25527454 [73]
*Usp22*: ubiquitin specific peptidase 22	Increase the abilities of proliferation, migration and invasion of glioma cells, and promote the growth and development of glioma	30223389 [74]
*Atp1a2*: ATPase, Na+/K+ transporting, alpha 2 polypeptide	Induce tumor progression and temozolomide resistance in glioma	27837435 [75]

## References

[1] Goffart N., Kroonen J., Rogister B. (2013). Glioblastoma-Initiating Cells: Relationship with Neural Stem Cells and the Micro-Environment. Cancers.

[2] Stupp R., E Hegi M., Mason W.P., Bent M.J.V.D., Taphoorn M.J., Janzer R.C., Ludwin S.K., Allgeier A., Fisher B., Bélanger K. (2009). Effects of radiotherapy with concomitant and adjuvant temozolomide versus radiotherapy alone on survival in glioblastoma in a randomised phase III study: 5-year analysis of the EORTC-NCIC trial. Lancet Oncol..

[3] Claes A., Idema A.J., Wesseling P. (2007). Diffuse glioma growth: A guerilla war. Acta Neuropathol..

[4] McCarthy D.J., Campbell K.R., Lun A.T.L., Wills Q.F. (2017). Scater: pre-processing, quality control, normalization and visualization of single-cell RNA-seq data in R. Bioinform..

[5] Zhang J., Guan M., Wang Q., Zhang J., Zhou T., Sun X. (2019). Single-cell transcriptome-based multilayer network biomarker for predicting prognosis and therapeutic response of gliomas. Brief Bioinform..

[6] Wang Y., Wu H., Yu T. (2017). Differential gene network analysis from single cell RNA-seq. J. Genet. Genom..

[7] Pina C., Teles J., Fugazza C., May G., Wang D., Guo Y., Soneji S., Brown J., Edén P., Ohlsson M. (2015). Single-Cell Network Analysis Identifies DDIT3 as a Nodal Lineage Regulator in Hematopoiesis. Cell Rep..

[8] Zhang B., Li H., Riggins R.B., Zhan M., Xuan J., Zhang Z., Hoffman E.P., Clarke R., Wang Y. (2008). Differential dependency network analysis to identify condition-specific topological changes in biological networks. Bioinform..

[9] Ali M., Del Sol A. (2018). Modeling of Cellular Systems: Application in Stem Cell Research and Computational Disease Modeling. Methods in Molecular Biology.

[10] Jardim V.C., Santos S.D.S., Fujita A., Buckeridge M.S. (2019). BioNetStat: A Tool for Biological Networks Differential Analysis. Front. Genet..

[11] Ideker T., Krogan N.J. (2012). Differential network biology. Mol. Syst. Boil..

[12] Islam S., Sarwar D.M. (2019). Identifying Brain Region Connectivity using Steiner Minimal Tree Approximation and a Genetic Algorithm.

[13] Xie J., Lu D., Li J., Wang J., Zhang Y., Li Y., Nie Q. (2018). Kernel differential subgraph reveals dynamic changes in biomolecular networks. J. Bioinform. Comput. Boil..

[14] Robinson M.D., McCarthy D.J., Smyth G.K. (2010). edgeR: A Bioconductor package for differential expression analysis of digital gene expression data. Bioinformatics.

[15] Benjamini Y., Hochberg Y. (1995). Controlling the False Discovery Rate: A Practical and Powerful Approach to Multiple Testing. J. R. Stat. Soc. Ser. B (Statistical Methodol..

[16] Cortese R., Hartmann O., Berlin K., Eckhardt F. (2008). Correlative gene expression and DNA methylation profiling in lung development nominate new biomarkers in lung cancer. Int. J. Biochem. Cell Boil..

[17] Wang Y., Navin N.E. (2015). Advances and applications of single-cell sequencing technologies. Mol. Cell.

[18] Bock C., Farlik M., Sheffield N.C. (2016). Multi-Omics of Single Cells: Strategies and Applications. Trends Biotechnol..

[19] Zhang P., Yang M., Zhang Y., Xiao S., Lai X., Tan A., Du S., Li S. (2019). Dissecting the Single-Cell Transcriptome Network Underlying Gastric Premalignant Lesions and Early Gastric Cancer. Cell Rep..

[20] Chen P., Li Y., Liu X., Liu R., Chen L. (2017). Detecting the tipping points in a three-state model of complex diseases by temporal differential networks. J. Transl. Med..

[21] Puniya B.L., Kulshreshtha D., Verma S.P., Kumar S., Ramachandran S. (2013). Integrated gene co-expression network analysis in the growth phase of Mycobacterium tuberculosis reveals new potential drug targets. Mol. BioSyst..

[22] Przulj N., Corneil D.G., Jurisica I. (2004). Modeling interactome: Scale-free or geometric?. Bioinform..

[23] Milenković T., Pržulj N. (2008). Uncovering Biological Network Function via Graphlet Degree Signatures. Cancer Informatics.

[24] Malod-Dognin N., Ban K., Pržulj N. (2017). Unified Alignment of Protein-Protein Interaction Networks. Sci. Rep..

[25] Milenković T., Ng W.L., Hayes W., Przulj N. (2010). Optimal Network Alignment with Graphlet Degree Vectors. Cancer Informatics.

[26] Smoot M.E., Ono K., Ruscheinski J., Wang P.L., Ideker T. (2011). Cytoscape 2.8: new features for data integration and network visualization. Bioinformatics.

[27] Jeong H., Mason S.P., Barabási A.L., Oltvai Z.N. (2001). Lethality and centrality in protein networks. Nat..

[28] Newman M.J. (2005). A measure of betweenness centrality based on random walks. Soc. Networks.

[29] Sabidussi G. (1966). The centrality index of a graph. Psychom..

[30] Bonacich P. (1987). Power and Centrality: A Family of Measures. Am. J. Sociol..

[31] Yu G., Wang L.G., Han Y., He Q.Y. (2012). clusterProfiler: An R Package for Comparing Biological Themes Among Gene Clusters. OMICS: A J. Integr. Boil..

[32] Yuan W., Li X., Liu L., Wei C., Sun D., Peng S., Jiang L. (2019). Comprehensive analysis of lncRNA-associated ceRNA network in colorectal cancer. Biochem. Biophys. Res. Commun..

[33] Chawla N.V., Bowyer K.W., Hall L.O., Kegelmeyer W.P. (2002). SMOTE: Synthetic Minority Over-sampling Technique. J. Artif. Intell. Res..

[34] Barabási A.L. (2009). Scale-free networks: A decade and beyond. Science.

[35] Zhang X.F., Ou-Yang L., Yan H. (2017). Incorporating prior information into differential network analysis using non-paranormal graphical models. Bioinform..

[36] Hougardy S. (2010). The Floyd–Warshall algorithm on graphs with negative cycles. Inf. Process. Lett..

[37] Darmanis S., Sloan S.A., Zhang Y., Enge M., Caneda C., Shuer L.M., Gephart M.G.H., Barres B.A., Quake S.R. (2015). A survey of human brain transcriptome diversity at the single cell level. Proc. Natl. Acad. Sci. USA.

[38] Darmanis S., Sloan S.A., Croote D., Mignardi M., Chernikova S., Samghababi P., Zhang Y., Neff N., Kowarsky M., Caneda C. (2017). Single-Cell RNA-Seq Analysis of Infiltrating Neoplastic Cells at the Migrating Front of Human Glioblastoma. Cell Rep..

[39] Chen Y., Liu P., Sun P., Jiang J., Zhu Y., Dong T., Cui Y., Tian Y., An T., Zhang J. (2019). Oncogenic MSH6-CXCR4-TGFB1 Feedback Loop: A Novel Therapeutic Target of Photothermal Therapy in Glioblastoma Multiforme. Theranostics.

[40] Doll S., Urisman A., Oses-Prieto J.A., Arnott D., Burlingame A.L. (2017). Quantitative Proteomics Reveals Fundamental Regulatory Differences in Oncogenic HRAS and Isocitrate Dehydrogenase (IDH1) Driven Astrocytoma. Mol. Cell Proteomics.

[41] Kina I., Sultuybek G.K., Soydas T., Yenmis G., Biceroglu H., Dirican A., Uzan M., Ulutin T. (2019). Variations in Toll-like receptor and nuclear factor-kappa B genes and the risk of glioma. Br. J. Neurosurg..

[42] Amodeo V.A.D., Betts J., Bartesaghi S., Zhang Y., Richard-Londt A., Ellis M., Roshani R., Vouri M., Galavotti S., Oberndorfer S. (2017). A PML/Slit Axis Controls Physiological Cell Migration and Cancer Invasion in the CNS. Cell Rep..

[43] Wang W., Zhao Z., Wu F., Wang H., Wang J., Lan Q., Zhao J. (2018). Bioinformatic analysis of gene expression and methylation regulation in glioblastoma. J. Neurooncol..

[44] Quick Q., Skalli O. (2010). Alpha-actinin 1 and alpha-actinin 4: contrasting roles in the survival, motility, and RhoA signaling of astrocytoma cells. Exp. Cell Res..

[45] De I., Steffen M.D., Clark P.A., Patros C.J., Sokn E., Bishop S.M., Litscher S., Maklakova V.I., Kuo J.S., Rodriguez F.J. (2016). CSF1 overexpression promotes high-grade glioma formation without impacting the polarization status of glioma-associated microglia and macrophages. Cancer Res..

[46] Hutterer M., Knyazev P., Abate A., Reschke M., Maier H., Stefanova N., Knyazeva T., Barbieri V., Reindl M., Muigg A. (2008). Axl and Growth Arrest Specific Gene 6 Are Frequently Overexpressed in Human Gliomas and Predict Poor Prognosis in Patients with Glioblastoma Multiforme. Clin. Cancer Res..

[47] Bonavia R., Inda M.M., Vandenberg S., Cheng S.Y., Nagane M., Hadwiger P., Tan P., Sah D.W.Y., Cavenee W.K., Furnari F.B. (2012). EGFRvIII promotes glioma angiogenesis and growth through the NF-kappa B, interleukin-8 pathway. Oncogene.

[48] Fan H.C., Chen C.M., Chi C.S., Tsai J.D., Chiang K.L., Chang Y.K., Lin S.Z., Harn H.J. (2019). Targeting Telomerase and ATRX/DAXX Inducing Tumor Senescence and Apoptosis in the Malignant Glioma. Int. J. Mol. Sci..

[49] Moraes L.A., Kar S., Foo S.L., Gu T., Toh Y.Q., Ampomah P.B., Sachaphibulkij K., Yap G., Zharkova O., Lukman H.M. (2017). Annexin-A1 enhances breast cancer growth and migration by promoting alternative macrophage polarization in the tumour microenvironment. Sci. Rep..

[50] Chen L., Lin L., Xian N., Zheng Z. (2019). Annexin A2 regulates glioma cell proliferation through the STAT3-cyclin D1 pathway. Oncol. Rep..

[51] Liu W.M., Huang P., Kar N., Burgett M., Muller-Greven G., Nowacki A.S., Distelhorst C.W., Lathia J.D., Rich J.N., Kappes J.C. (2013). Lyn Facilitates Glioblastoma Cell Survival under Conditions of Nutrient Deprivation by Promoting Autophagy. PLOS ONE.

[52] Halatsch M.-E., Löw S., Mursch K., Hielscher T., Schmidt U., Unterberg A., Vougioukas V.I., Feuerhake F. (2009). Candidate genes for sensitivity and resistance of human glioblastoma multiforme cell lines to erlotinib. J. Neurosurg..

[53] Offenhäuser C., Al-Ejeh F., Puttick S., Ensbey K.S., Bruce Z.C., Jamieson P.R., Smith F.M., Stringer B.W., Carrington B., Fuchs A.V. (2018). EphA3 Pay-Loaded Antibody Therapeutics for the Treatment of Glioblastoma. Cancers.

[54] Ma Y.F., Tang N., Thompson R.C., Mobley B.C., Clark S.W., Sarkaria J.N., Wang J.L. (2016). InsR/IGF1R Pathway Mediates Resistance to EGFR Inhibitors in Glioblastoma. Clin. Cancer Res..

[55] Dulken B.W., Leeman D.S., Boutet S.C., Hebestreit K., Brunet A. (2017). Single-Cell Transcriptomic Analysis Defines Heterogeneity and Transcriptional Dynamics in the Adult Neural Stem Cell Lineage. Cell Rep..

[56] Jaraíz-Rodríguez M., Tabernero M.D., González-Tablas M., Otero A., Orfao A., Medina J.M., Tabernero A. (2017). A Short Region of Connexin43 Reduces Human Glioma Stem Cell Migration, Invasion, and Survival through Src, PTEN, and FAK. Stem Cell Rep..

[57] Shao N.Y., Wang D.X., Wang Y., Li Y., Zhang Z.Q., Jiang Q., Luo W., Cao C. (2018). MicroRNA-29a-3p Downregulation Causes Gab1 Upregulation to Promote Glioma Cell Proliferation. Cell. Physiol. Biochem..

[58] Zhen Y.B., Chen X.F., Yan T., Zhao S.G. (2017). Expression of TAG1/APP signaling pathway in the proliferation and differentiation of glioma stem cells. Oncol. Lett..

[59] Puca F., Tosti N., Federico A., Kuzay Y., Pepe A., Morlando S., Savarese T., D’Alessio F., Colamaio M., Sarnataro D. (2019). HMGA1 negatively regulates NUMB expression at transcriptional and post transcriptional level in glioblastoma stem cells. Cell Cycle.

[60] Gouazé-Andersson V., Delmas C., Taurand M., Martinez-Gala J., Evrard S., Mazoyer S., Toulas C., Cohen-Jonathan-Moyal E. (2016). FGFR1 induces glioblastoma radioresistance through the PLCγ/Hif1α pathway. Cancer Res..

[61] Royet A., Broutier L., Coissieux M.M., Malleval C., Gadot N., Maillet D., Gratadou-Hupon L., Bernet A., Nony P., Treilleux I. (2017). Ephrin-B3 supports glioblastoma growth by inhibiting apoptosis induced by the dependence receptor EphA4. Oncotarget.

[62] Huang S.X., Zhao Z.Y., Weng G.H., He X.Y., Wu C.J., Fu C.Y., Sui Z.Y., Ma Y.S., Liu T. (2017). Upregulation of miR-181a suppresses the formation of glioblastoma stem cells by targeting the Notch2 oncogene and correlates with good prognosis in patients with glioblastoma multiforme. Biochem. Biophys. Res. Commun..

[63] Kim Y.W., Kwon C., Liu J.L., Kim S.H., Kim S. (2012). Cancer association study of aminoacyl-tRNA synthetase signaling network in glioblastoma. PLoS ONE.

[64] Gao W.Z., Guo L.M., Xu T.Q., Yin Y.H., Jia F. (2018). Identification of a multidimensional transcriptome signature for survival prediction of postoperative glioblastoma multiforme patients. J. Transl. Med..

[65] Zhang M., Pan Y., Qi X., Liu Y., Dong R., Zheng D., Chang Q., Zhang J., Fang W., Zhong Y. (2018). Identification of New Biomarkers Associated With IDH Mutation and Prognosis in Astrocytic Tumors Using NanoString nCounter Analysis System. Appl. Immunohistochem. Mol. Morphol..

[66] Qin W., Rong X., Dong J., Yu C., Yang J. (2017). miR-142 inhibits the migration and invasion of glioma by targeting Rac1. Oncol. Rep..

[67] Liu Y.Y., Chen M.B., Cheng L., Zhang Z.Q., Yu Z.Q., Jiang Q., Chen G., Cao C. (2018). microRNA-200a downregulation in human glioma leads to Galphai1 over-expression, Akt activation, and cell proliferation. Oncogene.

[68] Wang X., Prager B.C., Wu Q., Kim L.J.Y., Gimple R.C., Shi Y., Yang K., Morton A.R., Zhou W., Zhu Z. (2018). Reciprocal Signaling between Glioblastoma Stem Cells and Differentiated Tumor Cells Promotes Malignant Progression. Cell Stem Cell.

[69] Tseliou M., Al-Qahtani A., Alarifi S., Alkahtani S.H., Stournaras C., Sourvinos G. (2016). The Role of RhoA, RhoB and RhoC GTPases in Cell Morphology, Proliferation and Migration in Human Cytomegalovirus (HCMV) Infected Glioblastoma Cells. Cell. Physiol. Biochem..

[70] Wei Y., Wang F., Sang B., Xu Z., Yang D. (2019). Activation of KRas-ERK1/2 signaling drives the initiation and progression of glioma by suppressing the acetylation of histone H4 at lysine 16. Life Sci..

[71] Sun M.Z., Kim J.M., Oh M.C., Safaee M., Kaur G., Clark A.J., Bloch O., Ivan M.E., Kaur R., Oh T. (2013). Na⁺/K⁺-ATPase β2-subunit (AMOG) expression abrogates invasion of glioblastoma-derived brain tumor-initiating cells. Neuro-Oncology.

[72] Doan P., Musa A., Candeias N.R., Emmert-Streib F., Yli-Harja O., Kandhavelu M. (2019). Alkylaminophenol Induces G1/S Phase Cell Cycle Arrest in Glioblastoma Cells Through p53 and Cyclin-Dependent Kinase Signaling Pathway. Front. Pharmacol..

[73] Matsuda Y., Ishiwata T., Yoshimura H., Hagio M., Arai T. (2015). Inhibition of nestin suppresses stem cell phenotype of glioblastomas through the alteration of post-translational modification of heat shock protein HSPA8/HSC71. Cancer Lett..

[74] Liang J., Zhang X.L., Li S., Xie S., Wang W.-F., Yu R.-T. (2018). Ubiquitin-specific protease 22 promotes the proliferation, migration and invasion of glioma cells. Cancer Biomarkers.

[75] Yang J.K., Yang J.P., Tong J., Jing S.Y., Fan B., Wang F., Sun G.Z., Jiao B.H. (2017). Exosomal miR-221 targets DNM3 to induce tumor progression and temozolomide resistance in glioma. J. Neuro-Oncol..

[76] Modrek A.S., Prado J., Bready D., Dhaliwal J., Golub D., Placantonakis D.G. (2018). Modeling Glioma with Human Embryonic Stem Cell-Derived Neural Lineages. Advanced Structural Safety Studies.

